# Surprising Resolution of Life-Long Severe Generalized Hyperhidrosis Post-angioplasty and Stenting

**DOI:** 10.7759/cureus.52451

**Published:** 2024-01-17

**Authors:** Juwayria A Ahmed, Khudheeja A Ahmed, Mohammed Habeeb Ahmed

**Affiliations:** 1 Department of Research, KAAJ Healthcare, San Jose, USA; 2 Department of Cardiology, KAAJ Healthcare, San Jose, USA

**Keywords:** angioplasty and stenting, coronary artery disease, generalized hyperhidrosis, secondary hyperhidrosis, primary hyperhidrosis

## Abstract

Hyperhidrosis (HH) is a condition characterized by excessive sweating beyond thermoregulation needs. HH can be primary with no known etiology or secondary, as a symptom of underlying medical disease or a side effect of certain medications. Furthermore, HH can be focal, affecting one or a few body parts, or generalized, affecting the entire body. We present the case of a 49-year-old male with a history of primary generalized HH as well as coronary artery disease whose HH symptoms surprisingly resolved following coronary angioplasty and stenting. This unprecedented outcome of the procedure points to a potential association between HH and coronary artery disease, proposing potential management of HH through cardiovascular workup. In light of this result, we suggest that patients exhibiting generalized primary HH undergo a thorough comprehensive cardiovascular workup.

## Introduction

Hyperhidrosis (HH) is a skin disorder characterized by excessive sweating beyond what is necessary for homeostatic thermoregulation [1]. HH primarily affects people between the ages of 25 and 64, with 25 being the average age of onset [2]. HH affects about 3% of the general population, affecting both men and women equally [2]. However, due to the embarrassment of its presentation and limited awareness of its medical nature, HH is often underreported by patients, especially male patients, and a recent survey suggests that HH may affect at least 4.8% of the US population [2-4].

HH is classified as primary or secondary in origin. Within each category, HH is further classified as generalized (affecting the entire body), focal (affecting one or more body areas), regional, symmetric, or asymmetric based on anatomic distribution [3,5]. Primary HH, affecting approximately 93% of HH patients, is idiopathic in etiology and not known to be caused by underlying disease or medications [3]. This is thought to have a genetic basis since 35% to 56% of patients have a positive family history [3]. Primary HH typically begins in adolescence or between the ages of 14 and 25 years, but may also manifest in young children [2,4]. Primary HH is generally focal, bilateral, and symmetrical, affecting areas with a high density of eccrine sweat glands such as the axillae, palms, soles, and craniofacial regions [3,6]. Generalized primary HH is rare [4]. Among the diagnostic criteria for primary HH are episodes of sweating lasting at least seven days and excessive sweating for over six months [6].

Secondary HH can manifest as a symptom of various underlying medical conditions, including malignancy, infection, endocrine disorders like diabetes mellitus and hyperthyroidism, cardiovascular diseases like congestive heart failure, respiratory diseases, neurological disorders like stroke or Parkinson’s disease, and psychiatric disorders among others [3]. Secondary HH also commonly arises as a side effect of various medications, including certain antidepressants, antibiotics, antivirals, hypoglycemic agents, triptans, antipyretics, nonsteroidal anti-inflammatory drugs, adrenergic drugs, and even substances like alcohol, cocaine, or heroin, among many others [4]. Secondary HH is usually generalized but may rarely be focal or regional [3]. Physiologic conditions, such as elevated temperatures, fever, pregnancy, or menopause, can give rise to generalized secondary HH [2-4]. It is essential to rule out secondary causes before diagnosing primary HH. Indicators of secondary causes include an asymmetric, unilateral, or generalized distribution, nocturnal symptoms, onset after 25 years of age, and a negative family history [4].

The pathophysiology of primary HH is not well understood [5]. Of the human body’s approximately four million sweat glands, about three million are eccrine sweat glands which are believed to be responsible for primary HH [3]. Eccrine sweat glands are innervated by the cholinergic fibers of the sympathetic nervous system and normally function to regulate body temperature by secreting sweat [5]. They are distributed throughout the body with higher density in the feet and forehead, followed by the palms and face [5]. HH does not arise from an increased number of sweat glands, nor enlarged sweat glands, nor histopathological alterations in sweat glands [3,5]. Instead, it arises from the dysfunction of both the sympathetic and parasympathetic branches of the autonomic nervous system, leading to neurogenic hyperexcitability of the reflex circuits and the overstimulation of otherwise normal eccrine sweat glands [3,5]. An alternative theory proposes that since both emotional sweating and thermoregulatory sweating are triggered by sympathetic cholinergic nerves, HH may be attributed to abnormal central control of emotions [3].

HH can significantly compromise a patient's quality of life by causing substantial disruptions to daily tasks, social engagements, and work-related activities [7]. In fact, the impact of HH on the quality of life was found to be comparable to that experienced in severe psoriasis, rheumatoid arthritis, multiple sclerosis, and end-stage renal disease [8]. Additionally, the ability to cope with HH does not improve with time so it is paramount to diagnose and treat HH whenever possible [3]. Primary HH may be treated both nonsurgically or surgically and secondary HH is additionally treated by treating the underlying medical disease or discontinuing the responsible medication [6]. Topical treatments like aluminum salts effectively alleviate mild focal HH symptoms within three weeks but may cause skin irritation and require repeated applications every 24-48 hours [5]. Systemic approaches, primarily anticholinergic agents, are considered for generalized or non-responsive cases but are limited by potential adverse effects such as dry mouth, blurred vision, urinary retention, constipation, and tachycardia [5,6]. Iontophoresis, effective for focal palmoplantar HH, requires time-consuming sessions and may also cause skin irritation [5, 6]. Botulinum toxin A is a well-studied focal HH treatment but has limitations due to contraindications, pain, and cost [5]. Surgical treatments, including endoscopic thoracic sympathectomy, lumbar sympathectomy, excision of axillary tissue, and subcutaneous axillary curettage and liposuction, offer high efficacy rates but come with potential complications such as mild to severe compensatory HH in up to 86% of patients as well as gustatory sweating, neuralgia, Horner’s syndrome, and risk of hemothorax or pneumothorax [5].

## Case presentation

A 49-year-old male presented to the cardiologist’s office on March 31, 2023, with complaints of episodes of chest pain, occurring intermittently for the last few weeks, substernal in location and radiating to the left arm. The episodes occurred with no aggravating factors and were associated with palpitations, dizziness, tingling, and numbness of the left arm. He was also experiencing occasional episodes of shortness of breath.

The patient’s last angiogram done five and a half years ago on December 12, 2017 showed non-obstructive coronary artery disease involving the left anterior descending artery (LAD) and the diagonal artery. There was also a myocardial bridge seen in the mid to distal LAD. The left ventricular systolic function was normal, with an ejection fraction of 60% and 65%.

Past workup included an arterial ultrasound done on March 9, 2022, which showed biphasic and triphasic waveforms with no evidence of any hemodynamically significant arterial disease. An echocardiogram done on August 26, 2022 showed normal left ventricular size and systolic function, an ejection fraction (EF) of 60%-65%, mild to moderate left ventricular hypertrophy (LVH), E/A reversal mild thickened mitral valve (MV), mild thickened tricuspid valve (TV), and trace mitral regurgitation (MR). A 24-hour Holter done on July 14, 2022 showed sinus rhythm with a heart rate ranging from 73 beats per minute to 147 beats per minute with an average rate of 95 beats per minute. No arrhythmias were seen. An adenosine cardiolyte stress test done on July 14, 2022 showed a small, mild partially reversible inferior wall defect suggestive of mild ischemia. There was mild inferior wall hypokinesis with an EF of 45%.

The patient had a medical history of generalized primary HH known to the cardiologist for more than a decade. The patient was not undergoing any treatment by his primary care physician for this condition, and he was told that it was not a treatable condition. The patient also had COVID-19 in January of 2022. The patient was taking the following medications at the time: aspirin 81 mg, lisinopril/HCTZ 20/12.5 mg, metoprolol 50 mg twice daily, and loratadine once daily. The patient’s symptoms of chest pain, palpitations, dizziness, tingling and numbness, and shortness of breath continued in a follow-up visit on June 12, 2023. An echocardiogram done on this date showed sinus rhythm and was within normal limits. On a follow-up visit on July 17, 2023, the patient reported that his symptoms were occurring more frequently and worsening in intensity. The patient was scheduled for coronary angiography and possible angioplasty and stenting which was performed on July 18, 2023.

**Figure 1:**
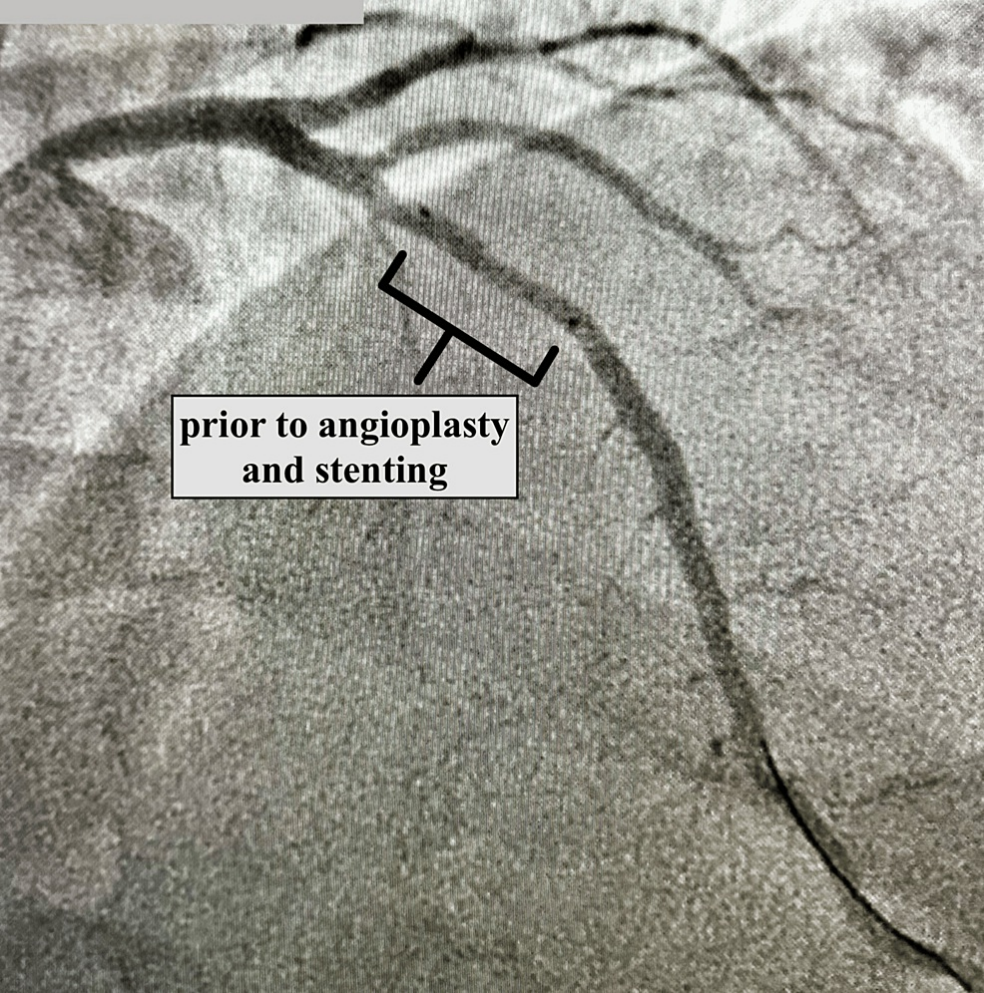
Angiography showing the site of coronary artery disease involving the LAD

**Figure 2:**
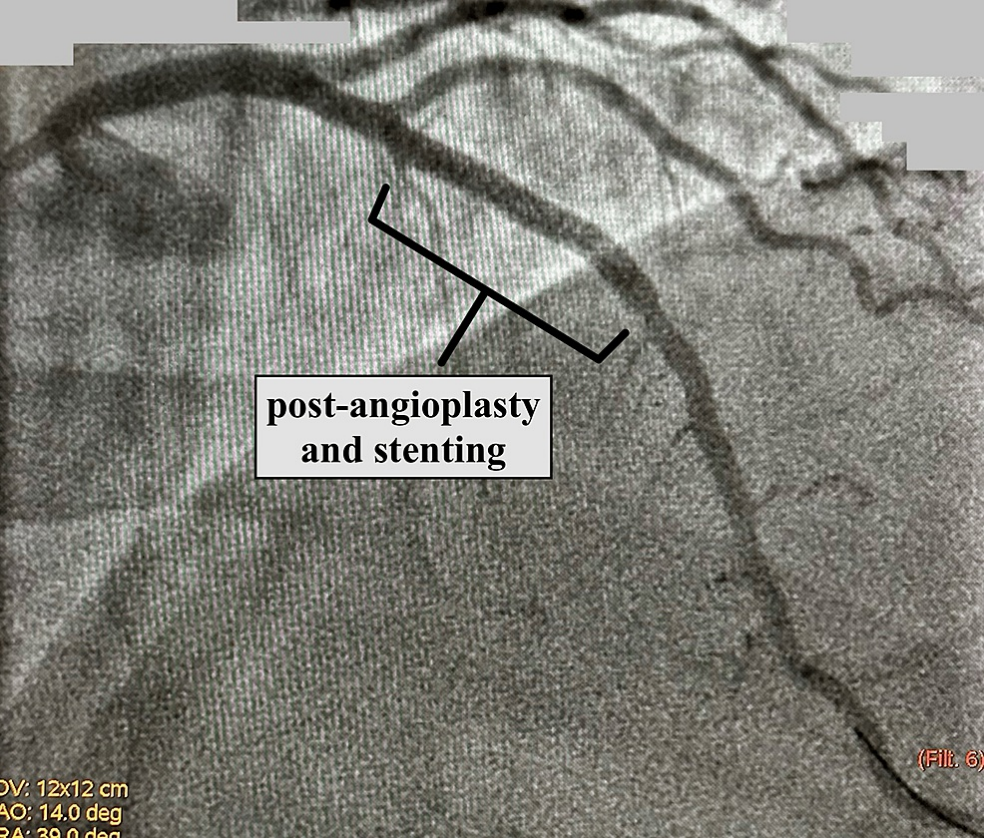
Angiography showing successful treatment of coronary artery disease involving the LAD

The angiography showed LAD had a diffuse long tapered area after the origin of the first diagonal artery and its stenosis was calculated to be between 68% and 72%, angiographically. The second diagonal artery had ostial/proximal 30% to 40% narrowing. The ventriculography showed global hypokinesis, more so of the distal half of the ventricle. The EF was calculated to be 28%. Intravascular ultrasonography of the LAD had shown two significant areas with 74%-76% stenosis. Successful angioplasty and stenting of the LAD were performed. Images taken from the angiography before and after stenting are shown in Figures 1, 2, respectively.

At the time of the follow-up post-angioplasty and stenting, the patient felt much better, with a resolution of the symptoms of angina. Remarkably, he was completely free of his HH for the first time in his life for as long as he could remember.

## Discussion

HH is a condition marked by excessive sweating, which can manifest either focally in one or more body parts or as a generalized phenomenon across the entire body [5]. Primary HH is usually focal and rarely generalized, while secondary HH is usually generalized and rarely focal [3,4]. Primary HH has no known etiology, whereas secondary HH is often a side effect of medications such as antidepressants, antibiotics, and antivirals, or is a symptom of various medical conditions, including malignancy, infection, endocrine disorders, cardiovascular diseases, respiratory diseases, neurological disorders, and psychiatric disorders [3,4].

We focus in particular on secondary HH as a symptom of cardiovascular disease. One review by Slavich et al. published in the American Journal of Cardiovascular Disease explored the relationship between HH and heart failure, proposing that unexplained sweating be perceived as a specific sign of heart failure [9]. The review cites a manuscript dating back to 1953 which found that episodes of excessive sweating lasting 15-30 minutes to a few hours can be anticipatory symptoms of severe cardiorespiratory crisis [9]. Another 1957 case report by Haugen presented the case of a patient hospitalized for heart failure with symptoms of profuse sweating but with reduced urinary output and no noticeable peripheral edema, suggesting an aldosterone-mediated mechanism that favored fluid elimination through skin sweating as a compensatory response [10]. Building on this hypothesis, a 1963 observational study on pediatric patients with congenital heart disease done by Morgan and Nadas similarly found a link between profuse sweating and the absence of edema. However, the authors proposed that neuro-vegetative hyperactivation (hyperactivation of the autonomic nervous system) rather than aldosterone-mediated hormonal regulation was the predominant pathophysiological mechanism for this phenomenon [11]. Consolidating these results, Slavich et al. hypothesize that in heart failure patients, the low cardiac output and subsequent reduced renal blood supply and diminished arterial baroreceptor pressure activate compensatory mechanisms, including the renin-angiotensin-aldosterone system (RAAS) and the adrenergic autonomic nervous system. This compensatory response, driven by fluid retention due to heart-kidney system decompensation, may lead to reactive HH, potentially serving as an early indicator of incipient decompensation and a preventive measure against manifest volume overload [9]. Additionally, sympathetic activation in heart failure patients was found to be associated with life-threatening cardiac arrhythmias and sudden death, prompting the authors to postulate that changes in sweating may be markers of adaptation or an early sign of decompensation in heart failure patients [9].

Furthermore, a single-center study done by Gokhroo et al. involving 11,695 patients with acute coronary syndrome (ACS) found that the presence of sweating with ACS symptoms (chest pain, shortness of breath, palpitations, etc.) predicts the probability of ST-elevation myocardial infarction (STEMI), even before clinical confirmation [12]. Moreover, the presence of sweating, whether in conjunction with typical or atypical angina, proves to be a more reliable predictor of STEMI compared to non-ST-elevation myocardial infarction (NSTEMI/ACS) [12]. A potential reason why excessive sweating is associated with STEMI and not with NSTEMI is the occurrence of transient hypotension triggered by acute myocardial stunning in STEMI [12]. This acute drop in blood pressure activates the sympathetic nervous system, leading to an immediate and intense sweating response [12]. The distinct absence of profuse sweating in NSTEMI can be attributed to the lack of transmural infarction and the absence of a comparable severe acute insult [12]. The authors propose that since both the sympathetic nervous system innervating sweat glands and the myocardial pain fibers originate in the thoracolumbar region, there may exist a cross-connection between these pathways. In parallel with the theory of referred pain, where sensations are perceived in an area distant from the actual stimulus source, the authors suggest that sweating could be a referred symptom. However, this proposed mechanism requires further scientific validation to establish its validity [12]. This study discusses ACS as a secondary cause for short-term secondary generalized HH rather than long-term primary HH but may still provide useful context to the potential link between generalized primary HH and cardiovascular disease.

In our case, we see a surprising case of HH, which was initially diagnosed as generalized primary HH and significantly resolved after coronary angioplasty and stenting, indicating a possible etiology and an avenue for its management. We noted that the patient’s quality of life has significantly improved following the procedure and resolution of HH symptoms. In light of this case, we recommend patients exhibiting generalized primary HH undergo thorough cardiovascular workup. Further research into the connections between such dermatological and cardiovascular conditions is recommended.

## Conclusions

In this report, we present a compelling case where symptoms associated with a diagnosis of generalized HH, hereto thought of as primary HH, resolved completely following coronary angioplasty and stenting. We have found no precedent in the literature for this occurrence. Consequently, this case contributes to the existing literature on the connection between coronary artery disease and HH and brings to light a potential etiological link and avenue for the management of HH. Considering the implications of this case, we advocate for a thorough cardiovascular workup for patients diagnosed with generalized primary HH and encourage further research to deepen the understanding of the emerging connection between HH and coronary artery disease.
